# Supplementary material for: S-Acylation of Proteins of Coronavirus and Influenza Virus: Conservation of Acylation Sites in Animal Viruses and DHHC Acyltransferases in Their Animal Reservoirs
Source: Pathogens. 2021 May 29;10(6):669. doi: 10.3390/pathogens10060669 (PMC8227752; doi:10.3390/pathogens10060669)
Supplement: Supplementary file 1 [file pathogens-10-00669-s001.zip › pathogens-1211795-supplementary.pdf]

Table S1: Amino acid sequences at the C-terminus of HA-subtypes from Flu A, HA from Flu B and HEF From Flu C and Flu D

	subtype	host	transmembrane domain											cytoplasmic tail										
Group-1	H1	A, H, P	V	S	L	G	A	I	S	F	W	M	C	S	N	G	S	L	Q	C	R	I	C	I
	H2	A, H, P	I	M	I	A	G	I	S	F	W	M	C	S	N	G	S	L	Q	C	R	I	C	I
	H5	A	I	M	V	A	G	L	S	L	W	M	C	S	N	G	S	L	Q	C	R	I	C	I
	H6	A	G	L	I	I	A	M	G	L	W	M	C	S	N	G	S	M	Q	C	R	I	C	I
	H8	A	I	L	I	A	G	G	L	I	L	G	M	Q	N	G	S	C	R	C	M	F	C	I
	H9	A	M	G	F	A	A	F	L	F	W	A	M	S	N	G	S	C	R	C	N	I	C	I
	H12	A	L	M	I	I	G	G	F	I	F	G	C	Q	N	G	N	V	R	C	T	F	C	I
	H11	A	A	I	I	M	G	F	I	F	W	A	C	S	N	G	S	C	R	C	T	I	C	I
	H13 gull	A	G	L	I	L	A	F	I	M	W	A	C	S	S	G	N	C	R	F	N	V	C	I
	H16 gull	A	G	L	I	L	A	F	I	M	W	A	C	S	S	G	N	C	R	F	N	V	C	I
	H17 bat	B	S	L	I	I	A	A	F	L	W	G	C	Q	K	G	S	I	Q	C	K	I	C	I
	H18 bat	B	L	I	I	L	A	A	I	E	W	G	C	F	K	G	N	L	Q	C	R	I	C	I
Group-2	H3	A, H, P,E	V	V	L	L	G	F	I	M	W	A	C	Q	K	G	N	I	R	C	N	I	C	I
	H4	A	A	L	L	L	A	F	I	L	W	A	C	Q	N	G	N	I	R	C	Q	I	C	I
	H7	A, E	A	I	A	M	G	L	V	F	I	C	I	K	N	G	N	M	R	C	T	I	C	I
	H10	A	A	V	I	M	G	L	V	F	F	C	L	K	N	G	N	M	R	C	T	I	C	I
	H14	A	A	L	I	L	G	F	V	L	W	A	C	Q	N	G	N	I	R	C	Q	I	C	I
	H15	A	A	I	A	M	G	L	I	F	M	C	V	K	N	G	N	L	R	C	T	I	C	I
	Flu B	H	L	M	I	A	I	F	I	V	Y	M	V	S	R	D	N	V	S	C	S	I	C	L
	toad	Am	A	I	T	I	A	A	G	I	M	F	L	C	K	N	G	R	C	T	I	C	I	
	eel	F	I	V	I	A	G	A	I	M	Y	M	G	K	S	G	N	I	R	C	N	I	C	L
	Flu C	H	L	A	A	L	V	I	S	G	I	A	C	R	T	K								
	Flu D	C, P	I	A	A	F	V	G	V	I	W	I	C	C	K	K								

Acylated cysteine residues are highlighted in yellow. Sequences are the consensus sequences calculated for each HA subtype and for HA of Flu B as described in Siche et al. H17 and H18 are viruses from bats.

H17: A/little yellow-shouldered bat/Guatemala/060/2010(H17N10). Accession number: AFC35438. H18: A/flat-faced bat/Peru/033/2010(H18N11); AGX84934:

Influenza C virus (C/Johannesburg/1/1966); UniProtKB - P07975.

Influenza D virus (D/bovine/France/2986/2012; NC_026952.

Toad: Wuhan asiatic toad influenza virus, unclassified Orthomyxovirus; AVM87632

Eel: Wuhan spiny eel influenza virus, unclassified Orthomyxovirus; AVM87624

Hosts: A: avian, H: human, P: pig, B: bat, E: equine, C: cattle, Am: amphibia, F: fish

Table S2: Accession numbers of HA sequences used to generate the phylogenetic tree in Fig.1

Virus name	Subtype	No
A/WSN/1933(H1N1)	H1	U08903.1
A/semi-palmated sandpiper/Brazil/43/1990(H2N1)	H2	CY005413.1
A/mallard duck/ALB/26/1976(H3N1)	H3	CY005943.1
A/mallard/ALB/47/1998(H4N1)	H4	CY004925.1
A/turkey/England/50-92/91(H5N1)	H5	EU636692.1
A/gray teal/Australia/1/1979(H6N1)	H6	CY014623.1
A/chicken/Rostock/45/1934(H7N1)	H7	CY077420.1
A/turkey/Ontario/6118/1968(H8N4)	H8	CY014659.1
A/mallard/Alberta/11/1991(H9N2)	H9	CY005990.1
A/mallard/ALB/5/1995(H10N1)	H10	CY005997.1
A/black duck/Ohio/194/1986(H11N1)	H11	CY017765.1
A/mallard/Interior Alaska/9BM1907R1/2009(H12)	H12	CY079894.1
A/herring gull/DE/475/1986(H13N2)	H13	CY005914.1
A/blue-winged teal/Guatemala/CIP049H106-62/2011(H14N6)	H14	KJ195668.1
A/sooty tern/Western Australia/2190/1983(H15N9)	H15	CY006033.1
A/herring gull/DE/712/1988(H16N3)	H16	CY005933.1
A/little yellow-shouldered bat/Guatemala/153/2009(H17N10)	H17	CY103876.1
A/Artibeus lituratus/Brazil/2301/2012(H18N11)	H18	MH682203.1
B/Lee/40	FluB HA	DQ792897.1
C/Ann Arbor/1/50	FluC HEF	NC_006310.2
D/bovine/Texas/72/2017	FluD HEF	MT636473.1

Table S3: Frequency of amino acids other than cysteine at position 50 of M2 of the indicated virus strains

**Virus host** HA-subtype	**Time period of collection date**	**Total M2 Sequences**	**Unique M2 Sequences**	**Percent of unique sequences not containing a Cys 50** Substituted by
**Avian M2**				
H1	1976-2018	945	158	**1,9%** Tyr, Ser
H2	1973-2018	541	109	**1,9%** Tyr
H3	1963-2019	2181	271	**2,2%** Tyr
H4	1956-2019	2012	224	**1,3%,** Tyr, Phe
H5	1995-2020	4208	845	**11,0%** Tyr
H5N1	1996-2019	2360	501	**10%,** Tyr
H6	1963-2018	1781	331	**8,5%**
H7	1902-2018	2163	344	**2,6%**
H7N9	2013-2017	623	94	**3,2%,** Tyr
H8	1967-2016	178	37	**11 %**
H9	1966-2019	2006	660	**12%**
H10	1949-2019	1281	144	**0,7%**
H11	1956-2019	767	135	**2,2%**
H12	1975-2018	349	62	**0**
H13	1977-2018	515	78	**2,5%**
H14	1982-2016	40	10	**0**
H15	1979-2015	10	1	**0**
H16	1975-2019	258	45	**0**
**Human M2**				
H1N1 „Spanish-Flu“	1918-1976	84	28	**0%**
H1N1 „Russian Flu“	1977-2008	1437	162	**74%,** Ser
H1N1 „Swine Flu“	2010-2020	11186	454	**0,2%,** Tyr
H2N2 „Asian Flu“	1957-1968	122	23	**0%**
H3N2 „Hongkong“	1968-2020	23232	905	**0,4%,** Tyr
H5 „bird Flu“	1997-2018	202	62	**24%,** Tyr, Phe
H7 „bird Flu“	1996-2018	127	36	**2,8%,** Tyr
**Porcine M2**				
H1N1	1930-2019	4050	934	**3,3%,** Tyr, Phe, Ser
H3N2	1978-2019	3030	620	**2,4%,** Tyr, Phe
H1N2	1980-2019	3174	676	**2,5%,** Tyr, Trp, Phe, Ser
**Equine M2**				
H7N7	1956-1977	15	7	**0%**
H3N8	1963-2017	200	45	**87%,** Phe
**Canine M2**				
H3N8	2003-2016	61	15	**87%,** Phe, Ser
H3N2	2006-2019	240	36	**0%**
**Bat M2**				
H17N10	2009-2010	2	2	**100%,** only Tyr
H18N11	2010-2011	2	2	**100%,** only Tyr

The NCBI database was searched for full-length M2 sequences (97 amino acids) present in a virus from a specific host, and having a specific HA (and partially NA) subtype as indicated. M2 sequences were aligned and the number of cysteines at position 50 calculated. The “time period of collection date” is the first and last year were the virus was isolated or sequenced.

Table S4: Amino acid sequences at the C-terminus of the spike protein of Coronaviruses

Genus	Subgenus	Species	Host	TMR	Cytoplasmic tail
Alpha	Pedacovirus	512/2005	Scoto. kuhlii	WYVWLAIVIALILVVSLLVF	CCISTGCCGCCGCCGSCFSGCCRGTKLQHYEPIEKVHVQ
	Tegacovirus	Insavc-1	Canis lupus	WYVWLLIGLVVIFCIPILLF	CCCSTGCCGCIGCLGSCCHSICSRGQFESYEPIEKVHVH
		FIPV	Felidae	WYVWLLIGLVVVFCIPLLLF	CCFSTGCCGCIGCLGSCCHSICSRRQFENYEPIEKVHVH
	Duvinacovirus	hCoV 229E	Homo sapiens	WWVWLCISVVLIFVVSMLLL	CCCSTGCCGFFSCFASSIRGCCESTKLPYYDVEKIHIQ
	Setracovirus	hCoV NL63	Homo sapiens	WWVWLIISVVFVVLLSLLVF	CCLSTGCCGCCNCLTSSMRGCCDCGSTKLPYYEFEKVHVQ
	Rhinacovirus	SADS	Sus scrofa	WAWWQWLLIFIALTLLAGLMLW	CCLATGCCGMCGCLAATCASCCDCRGTKLQSYEIEKVHVQ
Beta	Sarbecovirus	SARS2	Homo sapiens	WYIWLGFIAGLIAIVMVTIML	CCMTSCCSCLKGCCSCGSCCKFDEDDSEPVLKGVKLHYT
		RaTG13	R. affinis	WYIWLGFIAGLIAIIMVTIML	CCMTSCCSCLKGCCSCGSCCKFDEDDSEPVLKGVKLHYT
		Pangolin	M. javanica	WYIWLGFIAGLIAIIMVTIML	CCMTSCCSCLKGCCSCGSCCKFDEDDSEPVLKGVKLHYT
		SARS	Homo sapiens	WYVWLGFIAGLIAIVMVTILL	CCMTSCCSCLKGACSCGSCCKFDEDDSEPVLKGVKLHYT
		HKU3	R. sinicus	WYVWLGFIAGLIAIVMVTILL	CCMTSCCSCLKGACSCGSCCKFDEDDSEPVLKGVKLHYT
		Rp3/2004	R. spec	WYVWLGFIAGLIAIVMVTILL	CCMTSCCSCLKGACSCGSCCKFDEDDSEPVLKGVKLHYT
		279/2005	R. macrotis	WYVWLGFIAGLIAIVMVTILL	CCMTSCCSCLKGACSCGSCCKFDEDDSEPVLKGVKLHYT
		RsSHC014	R. sinicus	WYVWLGFIAGLIAIVMVTILL	CCMTSCCSCLKGACSCGSCCKFDEDDSEPVLKGVKLHYT
		Rs3367	R. sinicus	WYVWLGFIAGLIAIVMVTILL	CCMTSCCSCLKGACSCGSCCKFDEDDSEPVLKGVKLHYT
	Merbecovirus	MERS	Homo sapiens	WYIWLGFIAGLVALALCVFFI	LCCTGCGTNCMGKLKCNRCCDRYEEYDLEPHKVHVH
		HKU4	Tylo. pachypus	WYVWLGFIAGLVALLLCVFFL	LCCTGCGTSCLGKMKCKNCCDSYEEYDVEKIHVH
		Neo	Neo. capensis	WYIWLGFIAGLLALALCVFFI	LCCTGCGTSCLGKLKCNRCCDKYEEYDLEPHKIHVH
		HKU5	Pip. abramus	WYIWLGFIAGLVALALCVFFI	LCCTGCGTSCLGKLKCNRCCDSYDEYEVEKIHVH
		133/2005	Tylo.pachypus	WYIWLGFIAGLVALLLCVFFL	LCCTGCGTSCLGKMKCKNCCDSYEEYDVEKIHVH
	Embecovirus	hCoV HKU1	Homo sapiens	WYVWLLISFSFIIFLVLLFFI	CCCTGCGSACFSKCHNCCDEYGGHHDFVIKTSHDD
		hCoV OC43	Homo sapiens	WYVWLLICLAGVAMLVLLFFI	CCCTGCGTSCFKKCGGCCDDYTGYQELVIKTSHDD
		MHV	Mus musculus	WYVWLLIGLAGVAVCVLLFFI	CCCTGCGSCCFKKCGNCCDEYGGHQDSIVIHNISSHED
		Quebec	Bos taurus	WYVWLLIGFAGVAMLVLLFFI	CCCTGCGTSCFKICGGCCDDYTGHQELVIKTSHDD
	Nobecovirus	HKU9	R.leschenaulti	WYVWLAMIAGIVGLVLAVIML	MCMTNCCSCFKGMCDCRRCCGSYDSYDDVYPAVRVNKKRTV
Gamma	Igacovirus	IBV	Gallus gallus	VWLAIAFATIIFILILGWVFF	MTGCCGCCCGCFGIMPLMSKCGKKSSYYTTFDNDVVTEQ...
Delta	Andecovirus	HKU20	wigeon	WWVWLLIFLAICTFIIIVVTIFL	CTGCCGGCFGCFGGCCGLFSRVKHHEFSHLPVEEQDGSI...
	Herdecovirus	HKU19	Night heron	WYVWLAIALAFTGFVTILITIFL	CTGCCGGCFGCCGGCFGLFSKKIDPMRQYMNRYETPTSK...
	Buldecovirus	PDCoV	Sus scrofa	WYIWLAIALALIAFVTILITIFL	CTGCCGGCFGCCGGCFGLFSKKKRYTDDQPTPSFKFKEW
Unclassified		GCWSCoV	Tro. sinicus	WWVWLIISIVWTIFFLLILM	CCCFTGCCGICGCIARSARKRLDVEGGHFKYD

Cysteine residues are highlighted in yellow; …: indicate additional amino acids.

Accession numbers: SARS-CoV2 (sp|P0DTC2), SARS-CoV (sp|P59594), MERS-CoV (sp|K9N5Q8), Bat coronaviruses; HKU3 (SARS-like coronavirus HKU3(sp|Q3LZX1), HKU4 (Bat coronavirus HKU4 (sp|A3EX94), HKU5 (Bat coronavirus HKU5(sp|A3EXD0), HKU9 (Bat coronavirus HKU9 (sp|A3EXG6), Rp3/2004 (Bat coronavirus Rp3/2004(sp|Q3I5J5), 133/2005 (Bat coronavirus 133/2005(sp|Q0Q4F2), 512/2005 (Bat coronavirus 512/2005(sp|Q0Q466), 279/2005 (Bat coronavirus 279/2005 (sp|Q0Q475), RaTG13 (Bat coronavirus RaTG13) (tr|A0A6B9WHD3), RsSHC014 (Bat SARS-like coronavirus RsSHC014 (tr|U5WLK5), Rs3367 (Bat SARS-like coronavirus Rs3367 (tr|U5WHZ7), NeoCoV (Coronavirus Neoromicia/PML-PHE1/RSA/2011(AGY29650.2), Human coronaviruses; HKU1 (human coronaviruses HKU1-isolate N1) (sp|Q5MQD0), 229E (Human coronavirus 229E) (sp|P15423), NL63 (Human coronavirus NL63) (sp|Q6Q1S2) and OC43 (Human coronavirus OC43) (sp|P36334), Quebec (Bovine coronavirus Quebec strain) (sp|P25193), Insavc-1 (canine coronavirus Insavc-1 strain) (sp|P36300), IBV (Avian infectious bronchitis virus)(sp|P11223), FIPV (Feline infectious peritonitis virus) (sp|P10033), MHV (Murine hepatitis virus) (sp|P11224), Pangolin coronavirus (tr|A0A6G6A2R8), HKU20 (Wigeon coronavirus HKU20 (tr|H9BR25), HKU19 (Night heron coronavirus HKU19) (tr|H9BR17), SADS (Swine acute diarrhea syndrome coronavirus)(tr|A0A2P1G7F5), PDCoV (Porcine deltacoronavirus)(tr|A0A140ESF1), GCWSCoV (Guangdong Chinese water skink coronavirus) (AVM87574).

Table S5: Amino acid sequences at the C-terminus of the spike protein of newly discovered Coronaviruses with zoonotic potential

Genus	Subgenus	Species	Host	TMR	Cytoplasmic tail
Alpha	**Minunacovirus**	***Bat CoV 1***	Miniopterus sp.	WYVWLAIAVTLIIILVGPMLW	CCLATGCCGCCSCLVNSCSDCGGRRLQRYEIEKVHVQ
	**Pedacovirus**	***512/2005***	Scotophilus kuhlii	WYVWLAIVIALILVVSLLVF	CCISTGCCGCCGCCGSCFSGCCRGTKLQHYEPIEKVHVQ
	**Unclassified**	***BtKY22/2006***	Chaerephon sp.	WYVWLLIVVALTILAGLMLY	CCLATGCCGCCSCMTNTLDFRGRNLQRYEVEKVHVQ
Beta	**Sarbecovirus**	***SARS-2***	Homo sapiens	WYIWLGFIAGLIAIVMVTIML	CCMTSCCSCLKGCCSCGSCCKFDEDDSEPVLKGVKLHYT
		SARS	Homo sapiens	WYVWLGFIAGLIAIVMVTILL	CCMTSCCSCLKGACSCGSCCKFDEDDSEPVLKGVKLHYT
	**Embecovirus**	***Longquan Aa***	Apodemus agrarius	WYVWLLIGLAGVAVLVLLF...	CCCTGCGASCFKKCGSCCDDYGGHQDIVVKTSHDD
	**Merbecovirus**	***MERS***	Homo sapiens	WYIWLGFIAGLVALALCVFFI	LCCTGCGTNCMGKLKCNRCCDRYEEYDLEPHKVHVH
		SC2013	Vespertilio superans	WYIWLGFIAGLVALALCVF...	LCCTGCGTNCMGKLKCNRCCDKYDDYDVEPHKIHVH
	**Unclassified**	***BtKY24/2006***	Eidolon helvum	WYAWLGMIAGLVGLAMAVFMLC	CCMTNCCSGLKGICSCKQCQYDDYADVYPAVRVSGKRTV
**Unclassified**		***Rodent CoV***	Myodes rufocanus	WYVWLLIGLAGVAVLVLLF...	CCCTGCGSSCFKKCGGCCDDYGGHQELVIKTSHDD

Cysteine residues are highlighted in yellow: ….dots indicate incomplete sequences

Spike protein sequences of Coronaviruses; BtKY22/2006 (Chaerephon bat coronavirus/Kenya/KY22/2006) (tr|F1DB20), Longquan Aa (Longquan Aa mouse coronavirus) (tr|A0A096XNE4), BtKY24/2006 (Eidolon bat coronavirus/Kenya/KY24/2006) (tr|F1DAZ1), Rodent CoV (Rodent coronavirus) (tr|A0A2H4MXV6), Bat CoV 1 (Miniopterus bat coronavirus 1) (tr|B1PHJ5), SC2013 (BtVs-BetaCoV/SC2013) (tr|A0A023Y9K3), 512/2005 (Bat coronavirus 512/2005) (sp|Q0Q466)

Animal hosts and their taxonomy: *Chaerephon* sp. Is belonging to the bat *Molossidae*, *Miniopterus sp*. to the family *Miniopteridae*, *Eidolon helvum* to the family *Pteropodidae* and the bats *Scotophilus kuhlii* and *Vespertilio sinensis* belong to the family Vespertilionidae. The rodents *Apodemus agrarius* belongs to the family *Muridaea* and *Myodes rufocanus* to the family *Cricetidae*.

Table S6: Amino acid sequences at the C-terminus of the envelope protein of Coronaviruses

Genus	Subgenus	Species	Host	TMR	Cytoplasmic tail
Alpha	Pedacovirus	512/2005	Scoto. kuhlii	ILWLFVLFFVLVISITFVQLI	NLCFTCHRLCNNVVYKPVGKVYGVYKSYMRIQPLTSDIIQV
	Tegacovirus	Insavc-1	Canis lupus	IFWFLLIIILILFSIALLNII	KLCMVCCNLGRTVIIVPARHAYDAYKNFMQIRAYNPDEALLV
		FIPV	Felidae	FFWLLLIIILILFSIALLNVI	KLCMVCCNLGKTIIVLPARHAYDAYKTFMQTKAYNPDEAFLV
	Duvinacovirus	hCov 229E	Homo sapiens	LLWCVVLIVILLVCITIIKLI	KLCFTCHMFCNRTVYGPIKNVYHIYQSYMHIDPFPKRVIDF
	Setracovirus	hCoV NL63	Homo sapiens	ILWLLVMIFFFVLAMTFIKLI	QLCFTCHYFFSRTLYQPVYKIFLAYQDYMQIAPVPAEVLNV
	Rhinacovirus	SADS	Sus scrofa	...VLWLLVLILVLLVAITVIKLI	QLCFSCHRLMSNTIYIPVYNAYLVYKSYMEVEPCPIINV
Beta	Sarbecovirus	SARS2	Homo sapiens	VNSVLLFLAFVVFLLVTLAIL	TALRLCAYCCNIVNVSLVKPSFYVYSRVKNLNSSRVPDLLV
		RaTG13	R. affinis	...VLLFLAFVVFLLVTLAILTAL	RLCAYCCNIVNVSLVKPSFYVYSRVKNLNSSRVPDLLV
		Pangolin	M. javanica	...VLLFLAFVVFLLVTLAILTAL	RLCAYCCNIVNVSLVKPSFYVYSRVKNLNSSRVPDLLV
		SARS	Homo sapiens	VLLFLAFVVFLLVTLAILTAL	RLCAYCCNIVNVSLVKPTVYVYSRVKNLNSSEGVPDLLV
		HKU3	R. sinicus	VLLFLAFVVFLLVTLAILTAL	RLCAYCCNIVNVSLVKPTVYVYSRVKNLNSSEGVPDLLV
		Rp3/2004	R. spec	VLLFLAFVVFLLVTLAILTAL	RLCAYCCNIVNVSLVKPTVYVYSRVKNLNSSEGVPDLLV
		279/2005	R. macrotis	VLLFFAFVVFLLVTLAILTAL	RLCAYCCNIVNVSLVKPTVYVYSRVKNLNSSEGVPDLLV
		RsSHC014	R.sinicus	...VLLFLAFVVFLLVTLAILTAL	RLCAYCCNIVNVSLVKPTVYVYSRVKNLNSSQGVPDLLV
		Rs3367	R. sinicus	...VLLFLAFVVFLLVTLAILTAL	RLCAYCCNIVNVSLVKPTVYVYSRVKNLNSSEGVPDLLV
		MERS	Homo sapiens	FIFTVVCAITLLVCMAFLTAT	RLCVQCMTGFNTLLVQPALYLYNTGRSVYVKFQDSKPPLPP
		HKU4	Tylo. pachypus	FILTVVCAITLVVCLAILTAI	RLCVQCASGVNTLLFVPAFYIYNTGRNAYFKFQENRPPFPP
		Neo	Neo. capensis	FIFTVVCAITLLVCMAFLTAT	RLCVQCITGVNTLLVQPAVYMYNTGRSVYVKFQESKPPLPP...
		HKU5	Pip. abramus	FILSVVCAVTLVVCLAILTAI	RLCVQCVSGCHTLVFLPAVHIYNTGRAAYVKFQESHPPYPP
		133/2005	Tylo.pachypus	FILTVVCAITLLVCLAVLTAI	RLCVQCASGVNTLLFVPAFYIYNTGRNAYFKFQENRPPFPP
	Embecovirus	hCoV HKU1	Homo sapiens	LVLVLFCLISLIFVVAFLATI	KLCMQLCGFCNFFIISPSAYVYKRGMQLYKSYSEQVIPPTSD
		hCoV OC43	Homo sapiens	IFIVAICLLVTIVVVAFLATF	KLCIQLCGMCNTLVLSPSIYVFNRGRQFYEFYNDVKPPVLD
		MHV	Mus musculus	IFIFAVCLMVTIIVVAFLASI	KLCIQLCGLCNTLVLSPSIYLYDRSKQLYKYYNEEMRLPLLE
		Ontario	Bos taurus	IFIVAICLLVIIVVVAFLATF	KLCIQLCGMCNTLVLSPSIYVFNRGRQFYEFYNDVKPPVLD
	Nobecovirus	HKU9	R.leschenaulti	LVLIIICLLVVIVGCALLLIL	QFVFGVCGFVFKFVCKPTILVYNKFRNESLLNEREELL
Gamma	Igacovirus	IBV	Gallus gallus	NGSFLTALYIIVGFLALYLLG	RALQAFVQAADACCLFWYTWVVIPGAKGTAFVYKYTYGRK...
Delta	Andecovirus	HKU20	Wigeon	...PGSVVIAFLIVACCILLLLFI	KTCLAIFDCCSRSCTCVLDTVKPVYSFAYNLVPRFTPPYNP...
	Herdecovirus	HKU19	Night heron	...PAIYILVAVILLGCIVFLLLI	KTCLACCDFFTRTYTCILHTVKPIYVYLKPAPVVSKEDYIK...
	Buldecovirus	PDCoV	Sus scrofa	GQYIIAILVVTCIGVALLFI	NTCLACVKLFYKCYLGAAYLVRPIIVYYSKPNPVPEDEFVK...

Cysteine residues are highlighted in yellow; …: indicate additional amino acids

Accession numbers: SARS-CoV2 (sp|P0DTC4), SARS-CoV (sp|P59637), MERS-CoV (sp|K9N5R3), Bat coronaviruses; HKU3 (SARS-like coronavirus HKU3)(sp|Q3LZW9), HKU4 (Bat coronavirus HKU4) (sp|A3EX99), HKU5 (Bat coronavirus HKU5) (sp|A3EXD5), HKU9 (Bat coronavirus HKU9) (sp|A3EXG8), Rp3/2004 (Bat coronavirus Rp3/2004) (sp|Q3I5J3), 133/2005 (Bat coronavirus 133/2005) (sp|Q0Q4E8), 512/2005 (Bat coronavirus 512/2005) (sp|Q0Q464), 279/2005 (Bat coronavirus 279/2005) (sp|Q0Q473), RaTG13 (Bat coronavirus RaTG13) (tr|A0A6B9WFI6), RsSHC014 (Bat SARS-like coronavirus RsSHC014) (tr|U5WI28), Rs3367 (Bat SARS-like coronavirus Rs3367) (tr|U5WLL8), NeoCoV (Coronavirus Neoromicia/PML-PHE1/RSA/2011) (AIG13101.1), Human coronaviruses; HKU1 (human coronaviruses HKU1-isolate N1) (sp|Q5MQC8) , 229E (Human coronavirus 229E) (sp|P19741), NL63 (Human coronavirus NL63) (sp|Q6Q1S0) and OC43 (Human coronavirus OC43) (sp|Q04854), Ontario (Bovine coronavirus Ontario strain) (sp|P0C2Q6), Insavc-1 (canine coronavirus Insavc-1 strain) (sp|P36696), IBV (Avian infectious bronchitis virus)(sp|Q89894), FIPV (Feline infectious peritonitis virus) (sp|Q52PA5), MHV (Murine hepatitis virus) (sp|P0C2R0), Pangolin coronavirus (tr|A0A6G6A2K2), HKU20 (Wigeon coronavirus HKU20) (tr|H9BR26), and HKU19 (Night heron coronavirus HKU19) (tr|H9BR18), SADS (Swine acute diarrhea syndrome coronavirus)(tr|A0A2P1G6N3), PDCoV (Porcine deltacoronavirus)(tr|A0A0U2IVX8).

Table S7: Amino acid sequences at the C-terminus of the envelope protein of newly discovered Coronaviruses with zoonotic potential

Genus	Subgenus	Species	Host	TMR	Cytoplasmic tail
**Alpha**	**Minunacovirus**	***Bat CoV 1***	Miniopterus sp.	ILWLLFCVSVIIICCALIQLVQL...	QLCFICHRLCSNTVYKPVYKAYKIYQDYMQIEPLPVLNV
	**Pedacovirus**	***512/2005***	Scotophilus kuhlii	ILWLFVLFFVLVISITFVQLI	NLCFTCHRLCNNVVYKPVGKVYGVYKSYMRIQPLTSDIIQV
	**Unclassified**	***BtKY22/2006***	Chaerephon sp.	LNLLLWLMICCAVIVICNAVI	QLIFTCHRFCSSTVYTPVYAVYRIYQNYMRIDPLPVIDV
**Beta**	**Sarbecovirus**	***SARS2***	Homo sapiens	VLLFLAFVVFLLVTLAILTAL	RLCAYCCNIVNVSLVKPSFYVYSRVKNLNSSRVPDLLV
		SARS	Homo sapiens	VLLFLAFVVFLLVTLAILTAL	RLCAYCCNIVNVSLVKPTVYVYSRVKNLNSSEGVPDLLV
	**Embecovirus**	***Longquan Aa***	Apodemus agrarius	YVGQIILIVAICLLIIVIVVAA...	KLCIQLCGMCNTLVVSPSIYVYNRGRQFYKFYSDAKPP...
	**Merbecovirus**	***MERS***	Homo sapiens	FIFTVVCAITLLVCMAFLTAT	RLCVQCMTGFNTLLVQPALYLYNTGRSVYVKFQDSKPPLPP
		SC2013	Vespertilio superans	FIVNFFIFTVACAIILLVCMA...	RLCVQCAIGLNALLVQPAIYVYNTGRSVYVKFQESKPP...
	**Unclassified**	***BtKY24/2006***	Eidolon helvum	LITNILLLVIASLLFVIVGCAL...	GACGCLANIVCKPTIILYRKFKYESLLNEQEDII
**Unclassified**		***Rodent CoV***	Myodes rufocanus	YVGQIIFIFAICLFVIVVVVAFLA	TFKLCVQLCGICNTLVLSPSIYVYNRGRQFYEFYQD...

Cysteine residues are highlighted in yellow: … dots indicate incomplete sequences

Envelope protein sequences of Coronaviruses; BtKY22/2006 (Chaerephon bat coronavirus/Kenya/KY22/2006) (tr|F1DB22), Longquan Aa (Longquan Aa mouse coronavirus) (tr|A0A096XNF4), BtKY24/2006 (Eidolon bat coronavirus/Kenya/KY24/2006) (tr|F1DAZ3), Rodent CoV (Rodent coronavirus) (tr|A0A2H4N004), Bat CoV 1 (Miniopterus bat coronavirus 1) (tr|B1PHJ7), SC2013 (BtVs-BetaCoV/SC2013)(tr|A0A023Y9K8), 512/2005 (Bat coronavirus 512/2005)(sp|Q0Q464)

Animal hosts and their taxonomy: *Chaerephon* sp. Is belonging to the bat *Molossidae*, *Miniopterus sp*. to the family *Miniopteridae*, *Eidolon helvum* to the family *Pteropodidae* and the bats *Scotophilus kuhlii* and *Vespertilio sinensis* belong to the family Vespertilionidae. The rodents *Apodemus agrarius* belongs to the family *Muridaea* and *Myodes rufocanus* to the family *Cricetidae*.

Table S8: Amino acid differences between human DHHC2 and DHHC2 of animal hosts of Flu A

```
                      ....|....| ....|....| ....|....| ....|....| ....|....| ....|....|
                           5         15         25         35         45         55
Homo sapiens          ---------- --------MA PSGPGSS--- ARRRCRRVLY WIPVVFITLL LGWSYYAYAI
Sus scrofa            ---------- --------MA PSGPGS---- VRRRCRRVLY WIPVVFISLL LGWSYYAYAI
Gallus gallus         MRCGKMAAAA P------SAP AAGAAGGWGG VRRRCQRLLY WVPVLFISSI LCWSYYAYVT
Calidris pugnas       MRCEKMAAPP PP--PPLPLP PAGSAGTCMG LRRRCQRLLY WVPVLFISSI LCWSYYAYVT
Anas platyrhynchos    MRCGKMAAAP PPVAAAGAAA AAGAASGWGG VRRRCQRLLY WVPVLFISSI LCWSYYAYVT
                                                                     TMR1

                      ....|....| ....|....| ....|....| ....|....| ....|....| ....|....|
                           65        75         85         95         105        115
Homo sapiens          QLCIVSMENT GEQVVCLMAY HLLFAMFVWS YWKTIFTLPM NPSKEFHLSY AEKDLLEREP
Sus scrofa            QLCIVSMENI GEQVVCLIAY HLLFAMFVWS YWKTIFTLPM NPSKEFHLSY AEKELLEREP
Gallus gallus         QLCLLTMTNI GEKVVCLVAY HIFFMLFVWS YWKTIFTLPM NPSKEFHLSY SDKESLEREP
Calidris pugnas       QLCLLTMKNI GEKVVCLVAY HIFFMLFVWS YWKTIFTLPM NPSKEFHLSY SDKESLEREP
Anas platyrhynchos    QLCLLTMTNI GEKVVCLVAY HIFFMLFVWS YWKTIFTLPM NPSKEFHLSY SDKESLEREP
                                                  TMR2

                      ....|....| ....|....| ....|....| ....|....| ....|....| ....|....|
                           125       135        145        155        165        175
Homo sapiens          RGEAHQEVLR RAAKDLPIYT RTMSGAIRYC DRCQLIKPDR CHHCSVCDKC ILKMDHHCPW
Sus scrofa            RGEAHQEVLR RAAKDLPIYT RTMSGAIRYC DRCQLIKPDR CHHCSVCDKC ILKMDHHCPW
Gallus gallus         RGESQQEVLR RAAKDLPIYT RTMSGAIRYC DRCHLVKPDR CHHCSVCDKC ILKMDHHCPW
Calidris pugnas       RGESQQEVLR RAAKDLPIYT RTMSGAIRYC DRCHLVKPDR CHHCSICDKC ILKMDHHCPW
Anas platyrhynchos    RGESQQEVLR RAAKDLPIYT RTMSGAIRYC DRCHLVKPDR CHHCSVCDKC ILKMDHHCPW
                                                          Zinc-binding                 DHHC

                      ....|....| ....|....| ....|....| ....|....| ....|....| ....|....|
                           185       195        205        215        225        235
Homo sapiens          VNNCVGFSNY KFFLLFLAYS LLYCLFIAAT DLQYFIKFWT NGLPDTQAKF HIMFLFFAAA
Sus scrofa            VNNCVGFSNY KFFLLFLAYS LLYCLFIAAT DLQYFIKFWT NGLPDTQAKF HIMFLFFAAA
Gallus gallus         VNNCVGFSNY KFFLLFLAYS LLYCLFIAAT DLQYFIKFWT NGLPDTQAKF HIMFLFFAAA
Calidris pugnas       VNNCVGFSNY KFFLLFLAYS LLYCLFIAAT DLQYFIKFWT NGLPDTQAKF HIMFLFFAAA
Anas platyrhynchos    VNNCVGFSNY KFFLLFLAYS LLYCLFIAAT DLQYFIKFWT NGLPDTQAKF HIMFLFFAAA
                                           TMR3                                   TMR4

                      ....|....| ....|....| ....|....| ....|....| ....|....| ....|....|
                           245       255        265        275        285        295
Homo sapiens          MFSVSLSSLF GYHCWLVSKN KSTLEAFRSP VFRHGTDKNG FSLGFSKNMR QVFGDEKKYW
Sus scrofa            MFSVSLSSLF GYHCWLVSKN KSTLEAFRSP VFRHGTDKNG FSLGFSKNMR QVFGDEKKYW
Gallus gallus         MFSVSLSSLF GYHCWLVSKN KSTLEVFRAP IFHHRTDKNG FSLGFSKNLR QVFGDEKKYW
Calidris pugnas       MFSVSLSSLF GYHCWLVSKN KSTLEVFRAP IFRHRTDKNG FSLGFSKNLR QVFGDEKKYW
Anas platyrhynchos    MFSVSLSSLF GYHCWLVSKN KSTLEVFRAP IFRHRTDKNG FSLGFSKNLR QVFGDEKKYW
                                                  TTXE                       amphiphilic helix

                      ....|....| ....|....| ....|....| ....|....| ....|....| ....|....|
                           305       315        325        335        345        355
Homo sapiens          LLPIFSSLGD GCSFPTCLVN QDPEQASTPA GLNSTAKNLE NHQFPAKPLR ESQSHLLTDS
Sus scrofa            LLPIFSSLGD GCSFPTCLVN QDPEQPSTPA GLNSSSKNPE NHQFPAKPLR ESQSHLLTDS
Gallus gallus         LLPVFSSLGD GCSFPTCLVN QDPEQASTPG GLNSTCKN-E NHLFPAKPLR DSQSHLLTDT
Calidris pugnas       LVPVFSSLGD GCSFPTCLVN QDPEQASTPS ALNSTSKN-E NHLFPAKPLR DSQSHLLTDT
Anas platyrhynchos    LLPVFSSLGD GCSFPTCLVN QDPEQASTPG GLNSTSKN-E SLLFPAKPLR DSQSHLLTDT
                                  End of model              unstructured

                      ....|....| ....|....| ....|...
                           365       375        385
Homo sapiens          QSWTESSINP GKCKAGMSNP ALTMENET
Sus scrofa            QSWTESSTNP GKGKSGMSNP ALTMENET
Gallus gallus         HSWSDIGAKA EKGKVGMSNP ALTMENET
Calidris pugnas       PSWSETSGKA EKGKVGMSNP ALTMENET
Anas platyrhynchos    QSWSDISAKA EKGKIGMSNP ALTMENET
```

Amino acid alignment of DHHC2 of humans (homo sapiens), pigs (sus scrofa) and three avian species, chicken (gallus gallus), sandpiper (Calidris pugnas) and mallard duck (Anas platyrhynchos). The transmembrane regions are highlighted in grey, individual residues that contact the acyl chain in the cavity are highlighted in green and the two (S and T) that seal the cavity are highlighted in pink. The amphiphilic helix following TMR4 is also highlighted in grey. Cys and His residues that coordinate zinc ions, the DHHC motif and the TTEX motiv (which is STLE in DHHC2) are highlighted in light blue. LVN is the end of the modeled structure and underlined is the part of the C-terminal region predicted to be unstructured.
Amino acid differences to human DHHC2 are highlighted in yellow in the sequence of the respective species and the non-conservative exchanges labeled in the 3D-structure of DHHC2 are in red.

Table S9: Amino acid differences between human DHHC15 and DHHC15 of animal hosts of Flu A

```
              ....|....| ....|....| ....|....| ....|....| ....|....| ....|....|
                      5         15         25         35         45         55
Homo sapie    MRRGWKMALS GGLRCCRRVL SWVPVLVIVL VVLWSYYAYV FELCLVTVLS PAEKVIYLIL
Sus scrofa    MRRGWKMALS GGLRCCRRIL SWVPVLVIVL VVLWSYYAYV FELCLVTVLS PAEKVIYLIF
Gallus gal    ------MALS RGLRCCQRAF AWVPVLIITL VVLWSYYAYV CELCLMTLSN PAEKVAYLII
Calidris p    ------MALS RGLRCCQRAF AWLPVLIITL VVLWSYYAYV CELCLVTLSN PVEKVAYLII
Anas platy    ------MALS RGLRCCQRAF AWLPVLIIAL VVLWSYYAYV CELCLVTLSN PVEKVAYLII
                                            TMR1                             TMR2

              ....|....| ....|....| ....|....| ....|....| ....|....| ....|....|
                     65         75         85         95        105        115
Homo sapie    YHAIFVFFTW TYWKSIFTLP QQPNQKFHLS YTDKERYENE ERPEVQKQML VDMAKKLPVY
Sus scrofa    YHAIFVFFTW TYWKSIFTLP QQPNHKFHLS YTDKERYENE ERPEVQKQML VDMAKKLPVY
Gallus gal    FHILFVLFVW TYWKSIFTLP VQPGKKYHMS YADKERYENE ERPEVQRQIL AEIARKLPVY
Calidris p    FHILFVLFVW TYWKSIFTLP VQPGKKYHMS YADKERYENE ERPEVQRQIL AEMARKLPVY
Anas platy    FHILFVLFVW TYWKSIFTLP VQPGKKYHMS YADKERYENE ERPEVQRQIL AEIARKLPVY
                              Cysteine-rich domain

              ....|....| ....|....| ....|....| ....|....| ....|....| ....|....|
                    125        135        145        155        165        175
Homo sapie    TRTGSGAVRF CDRCHLIKPD RCHHCSVCAM CVLKMDHHCP WVNNCIGFSN YKFFLQFLAY
Sus scrofa    TRTGSGAVRF CDRCHLIKPD RCHHCSVCAM CVLKMDHHCP WVNNCIGFSN YKFFLQFLAY
Gallus gal    TRTGSGGIRF CDRCQLIKPD RCHHCSVCAI CVLKMDHHCP WVNNCIGFSN YKFFLLFLAY
Calidris p    TRTGSGGIRF CDRCQLIKPD RCHHCSVCAI CVLKMDHHCP WVNNCIGFSN YKFFLLFLAY
Anas platy    TRTGNGGIRF CDRCQLIKPD RCHHCSVCAI CVLKMDHHCP WVNNCIGFSN YKFFLLFLAY
                         Zinc-binding                DHHC                    TMR3

              ....|....| ....|....| ....|....| ....|....| ....|....| ....|....|
                    185        195        205        215        225        235
Homo sapie    SVLYCLYIAT TVFSYFIKYW RGELPSVRSK FHVLFLLFVA CMFFVSLVIL FGYHCWLVSR
Sus scrofa    SVLYCLYIAT TVFSYFIKYW RGELPSVRSK FHVLFLLFVA CMFFVSLVIL FGYHCWLVSR
Gallus gal    SLLYCLYIAA TVFKYFIKYW TGELTNGRSK FHILFLLFLA VMFFVSLMFL FGYHCWLVSR
Calidris p    SLLYCLYIAA TVFKYFIKYW TGELTNGRSK FHILFLLFVA IMFFVSLMFL FGYHCWLVSR
Anas platy    SLLYCLYIAA TVFKYFIKYW TGELTNGRSK FHVLFLLFVA VMFFVSLMFL FGYHCWLVSR

              ....|....| ....|....| ....|....| ....|....| ....|....| ....|....|
                    245        255        265        275        285        295
Homo sapie    NKTTLEAFCT PVFTSGPEKN GFNLGFIKNI QQVFGDKKKF WLIPIGSSPG DGHSFPMRSM
Sus scrofa    NKTTLEAFCT PVFTSGPEKN GFNLGFIKNI QQVFGDNKKF WLIPIGSSPG DGHSFPMRSM
Gallus gal    NRSTLEAFST PVFQNGPDKN GFNLGFVKNL QQVFGEEKKL WLLPIASSQG DGHFFPMRAL
Calidris p    NRSTLEAFSA PVFQNGPDKN GFNLGFVKNL QQVFGEEKKL WLLPIASSQG DGHFFPMRAL
Anas platy    NRSTLEAFSA PVFQNGPDKN GFNLGFVKNL QQVFGEEKKL WLLPIASSQG DGHFFPMRAL

              ....|....| ....|....| ....|....| ....|...
                    305        315        325        335
Homo sapie    NESQNPLLAN EETWEDNEDD NQDYPEG-SS SLAVETET
Sus scrofa    NESQNPLLAN EEPWEDNEDD NRDYPEG-SS SLAVETET
Gallus gal    CEAQNPLLSN EEQWEDDGID EEPHDSGEAA SLAIERET
Calidris p    CEAQNPLLAN EEQWEDDGID EEPHDSGEAS SLAIERET
Anas platy    CEAQNPLLAN EEQWEDDGID EEPHDSTEAS SLAIERET
```

Amino acid alignment of DHHC15 of humans (homo sapiens), pigs (sus scrofa) and three avian species: chicken (gallus gallus), sandpiper (Calidris pugnas) and mallard duck (Anas platyrhynchos). The transmembrane region is highlighted in grey, individual residues that contact the acyl chain in the cavity are highlighted in green and the two (S, Y) that seal the cavity are highlighted in pink. The amphiphilic helix following TMR4 is also highlighted in grey. Cys and His

residues that coordinate zinc ions, the DHHC motif and the TTEX motif are highlighted in light blue in the human sequence. NESQ is the end of the resolved structure. Amino acid differences to human DHHC15 are highlighted in yellow in the sequence of the respective species and the non-conservative exchanges labeled in the 3D-structure of DHHC15 are in red.

Table S10: Amino acid differences between human DHHC8 and DHHC8 from animal hosts of Flu A

```
            ....|....| ....|....| ....|....| ....|....| ....|....| ....|....|
                    5         15         25         35         45         55
Homo sapie  MPRSPGTRLK PAKYIPVATA AALLVGSSTL FFVFTCPWLT RAVSPAVPVY NGIIFLFVLA
Sus scrofa  MPRSPGTRLK PAKYIPVATA AALLVGSSTL FFVFTCPWLT RAVSPAVPVY NGILFLFVLA
Gallus gal  MPSSTGKRFK PTKYIPVSTA AALLVGSTTL FFVFTCPWLT KAISPAIPVY NGLVFLFVLA
Calidris p  ---------- ---------- ---------- ---------- ---------- ----------
Anas platy  MPSSTGKRFK PTKYIPVSTA AALLVGSTTL FFVFTCPWLT KAISPAIPVY NGLVFLFVLA
                                    TMR1                               TMR2

            ....|....| ....|....| ....|....| ....|....| ....|....| ....|....|
                   65         75         85         95        105        115
Homo sapie  NFSMATFMDP GVFPRADEDE DKEDDFRAPL YKNVDVRGIQ VRMKWCATCH FYRPPRCSHC
Sus scrofa  NFSMATFMDP GVFPRADEDE DKEDDFRAPL YKNVDVRGIQ VRMKWCATCH FYRPPRCSHC
Gallus gal  NFSMATFMDP GVFPRADEDE DKDDDFRAPL YKNVEIKGIQ VRMKWCATCH FYRPPRCSHC
Calidris p  ---MATFMDP GVFPRADEDE DKDDDFRAPL YKNVEIKGIQ VRMKWCATCH FYRPPRCSHC
Anas platy  NFSMATFMDP GVFPRADEDE DKDDDFRAPL YKNVEIKGIQ VRMKWCATCH FYRPPRCSHC
                             Cysteine-rich domain       zinc-binding

            ....|....| ....|....| ....|....| ....|....| ....|....| ....|....|
                  125        135        145        155        165        175
Homo sapie  SVCDNCVEDF DHHCPWVNNC IGRRNYRYFF LFLLSLSAHM VGVVAFGLVY VLNHAEGLGA
Sus scrofa  SVCDNCVEDF DHHCPWVNNC IGRRNYRYFF LFLLSLSAHM VGVVAFGLVY VLNHAEGLGA
Gallus gal  SVCDNCVEDF DHHCPWVNNC IGRRNYRYFF LFLLSLSTHM VGVFTFGLIF VLNHMEKLGA
Calidris p  SVCDNCVEDF DHHCPWVNNC IGRRNYRYFF LFLLSLSTHM VGVFTFGLIF VLNHMEKLGA
Anas platy  SVCDNCVEDF DHHCPWVNNC IGRRNYRYFF LFLLSLSTHM VGVFTFGLIF VLNHMEKLGA
                      DHHC                       TMR3
            ....|....| ....|....| ....|....| ....|....| ....|....| ....|....|
                  185        195        205        215        225        235
Homo sapie  AHTTITMAVM CVAGLFFIPV IGLTGFHVVL VTRGRTTNEQ VTGKFRGGVN PFTRGCCGNV
Sus scrofa  AHTTITMAVM CVAGLFFIPV IGLTGFHVVL VTRGRTTNEQ VTGKFRGGVN PFTRGCYGNV
Gallus gal  AHTTITMAVM CVAGLFFIPV IGLTGFHIVL VARGRTTNEQ VTGKFRGGVN PFTRGCCGNV
Calidris p  AHTAITMAVM CVAGLFFIPV IGLTGFHIVL VARGRTTNEQ VTGKFRGGVN PFTRGCCGNV
Anas platy  AHTTITMAVM CVAGLFFIPV IGLTGFHIVL VARGRTTNEQ VTGKFRGGVN PFTRGCCGNV
                             TMR4                  TTXE             amphiphilic

            ....|....| ....|....| ....|....| ....|....| ....|....| ....|....|
                  245        255        265        275        285        295
Homo sapie  EHVLCSPLAP ---------- -----RYVVE PPRLPLAVSL KPPFLRPELL DRAAPLKVKL
Sus scrofa  EHVLCSPLAP ---------- -----RYVVE PPRLPLAVRL KPPFLRPELL ER-APLKVKL
Gallus gal  EHVLCSPLAP SLLHLPLQGS CFMITRYIVE P-KKKLAVSV KPPFLRPDLS ER--QITVKI
Calidris p  EHVLCSPLAP ---------- -----RYIVE P-KKKQAVSV KPPFLRPDLS ER--QITVKI
Anas platy  EHVLCSPLAP S-LHLPPQGS CFMLTRYIVE P-KKKQAVSV KPPFLRPDLS ER--QITVKI
               helix

            ....|....| ....|....| ....|....| ....|....| ....|....| ....|....|
                  305        315        325        335        345        355
Homo sapie  SDNGLKAGLG RSKSKGSLDR LDEKPLDLGP PLPPKIEAGT FSSDLQTPRP GSAESALSVQ
Sus scrofa  SDNGLKAGLG RSKSKGSLDR LDEKPLDLGP PLPPKAEAST FGSDLQTPRP GSAESALSAH
Gallus gal  SDNGIQANLN RSKSKISLEG LEDKGMDVQP PLPPKGDQSK Y-SELKGQLG TSEESGLSPK
Calidris p  SDNGIQANLN RSKSKISLEG LEDTSMDVQP PLPPKGDPSK Y-SELKGQLG TGEEGSLSPK
Anas platy  SDNGIQANLN RSKSKISLEG LEDKSMDVQP PLPPKGDQSK Y-SELKGQLG TCEEGGLSPK

            ....|....| ....|....| ....|....| ....|....| ....|....| ....|....|
                  365        375        385        395        405        415
Homo sapie  RTSPPTPAMY KFRPAFPTGP KVPFCGPGEQ VPGPDSLTLG ---DDSIRSL DFVSEPSLDL
Sus scrofa  RTSPPTPAMY KFRPNFPTGP KAPFCGPGEQ VTGTDSLTLG ---DDSIHSL DFASEPSLDL
Gallus gal  LISPPTPAMY KYRPAFSNNP KVHYHAAAEQ ITLQEGHSQG ALIEEDGRSL DYQSEPSLDI
Calidris p  LISPPTPAMY KYRPAFSNNP KVHYHATAEQ ITMQEGHSQG ALIEEDGRSL DYQSEPSLDI
Anas platy  LISPPTPAMY KYRPAFSNNP KVHYHAAAEQ ITMQEGHSQG ALIEEDGGSL DYQSEPSLDI

            ....|....| ....|....| ....|....| ....|....| ....|....| ....|....|
                  425        435        445        455        465        475
Homo sapie  PDYGPGGLHA AYPPSPPLSA SDAFSGALRS LSLKASSRRG GDHVALQPLR SEGGPPTPHR
Sus scrofa  PDYPPGGLHA AYPPSPPLSA ADTFSGALRS LSLKAASRRG GDHVALQPLR SEGGPPTPHR
Gallus gal  PSYRKSSLHK TYQSSP--LQ IDSFAINSRS LSLKSAGRRG TDKVPLHPIK SEGAASTPYK
Calidris p  PSYRKSSLHK TYQSSP--LQ IDSFAINSRS LSLKSAGRRG TEKVPLHPIK SEGAASTPYK
Anas platy  PSYRKSSLHK TYQSSP--LQ IDSFAINSRS LSLKSAGRRG TDKVPLHPIK SEGATSTPYK
```

```
              ....|....| ....|....| ....|....| ....|....| ....|....| ....|....|
                   485        495        505        515        525        535
Homo sapie    SIFAPHALPN RNGSLSYDSL LNPGSPGGHA CPAHPAVGVA GYHSPYLHPG AT----GDPP
Sus scrofa    GLFAPHALPN RNGSLSYDSL LNPGSPGGHA CPAHSSAGVP GYRSPYLHTG AV----GDPP
Gallus gal    SIFSPNSLSN RNGSLSYDSL LNPMSPSGRK CVTHSAVSSV GYHSPYLSAK MCHLRGSELQ
Calidris p    SIFSPNSLSN RNGSLSYDSL LNPMSPSGRK CITHSAVSSV GYHSPYLSAK MCHLRGSELQ
Anas platy    SIFSPNSLSN RNGSLSYDSL LNPMSPSGRK CIAHSAVSSV GYHSPYLSAK MCHLRGSELQ

              ....|....| ....|....| ....|....| ....|....| ....|....| ....|....|
                   545        555        565        575        585        595
Homo sapie    RPLPRSFSPV LG---PRPRE PSPVRYDNLS RTIMASIQER KDREERERLL RSQADSLFGD
Sus scrofa    RPPPRSFSPV LG---PRPRE PSPVRYDNLS RTIMASIQER KDREERERLL RSQADSLFGD
Gallus gal    RQPPQSFSPV LGGPAPHQRD PSPVRYDNLS KTIMASIQER KEMEEREKLL HSHPDSVFAD
Calidris p    RQPPQSFSPV LGGPAPHQRD PSPVRYDNLS KTIMASIQER KEMEEREKLL HSHPDSVFAD
Anas platy    RQPPQSFSPV LGGPAPHQRD PSPVRYDNLS KTIMASIQER KEMEEREKLL HSHPDSVFAD

              ....|....| ....|....| ....|....| ....|....| ....|....| ....|....|
                   605        615        625        635        645        655
Homo sapie    SGVYDAPSSY SLQQASVLSE GPRGPALRYG SRDDLVAGPG FGGARNPALQ TSLSSLSSSV
Sus scrofa    SGVYDAPSSY SLQQASVLSE GPRGPVVRFG SRDDLVAGPG FGGARNPALQ ASLSSLSSTV
Gallus gal    SGVYDTPSSY SLQQVSTLSE DPRSMAMRYG SRDNLMAATS FS-TRNPILQ SSVSSLSSAM
Calidris p    SGVYDTPSSY SLQQVSTLSE DPRSMAMRYG SRDNLMAATS FS-TRNPILQ SSVSSLSSAM
Anas platy    SGVYDTPSSY SLQQVSTLSE DPRSMAMR-- ---------- ---------- ----------

              ....|....| ....|....| ....|....| ....|....| ....|....| ....|....|
                   665        675        685        695        705        715
Homo sapie    SRAPRTSSSS LQADQASSN- ----APGPRP SSGSHRSPAR QGLPSPPGTP HSPSYAGPKA
Sus scrofa    SRAPRTSSSS LQADLANSS- ----APGARP ASGSHRSPAR QVPPSPPGTP RSPSYAGPKA
Gallus gal    TRAPRTSTTS LQADLANNNV QTHQALQGRV SNGSYKSPGH QVPSSPTGMP RSPSYGGPKA
Calidris p    TRAPRTSTTS LQADLANNNV QSHQALQGRV SNGSFKSPGH QVPSSPTGMP RSPSYGGPKA
Anas platy    ---------- ---------- ---------- ---------- ---------- ----------

              ....|....| ....|....| ....|....| ....|....| ....|....| ....|....|
                   725        735        745        755        765        775
Homo sapie    VAFIHTDLPE PPPSLTVQRG RIGTCTRGWG RRGQPWVPPG LHLCHLGRPE DRPPLRAPWS
Sus scrofa    VAFIHTDLPE PPPSLAVQRG RIGTCSRGWG RRGQPRVPPS LHLCHLGLPE DRPPLRAPWS
Gallus gal    ISFVNTVEIT EVQSVGAQRD DIQMKTPHSK INGQP----- KGISRLG--S TSSSQGTPVS
Calidris p    VSFVNTVEIT EVQSVGAQRD DMQLKTPHSK INGQP----- KGISRLG--S TSSSQGTPVS
Anas platy    ---------- ---------D DMQLKTPHSK INGQP----- KGISRLG--S TSSSQGTPVS

              ....|....| ....|....| ....|....| ....|....| ....|....| ....|....|
                   785        795        805        815        825        835
Homo sapie    QAAGAPPRGA MCRLHLAASS LFPSLSGP-- ---------- ---------- ----------
Sus scrofa    PATGAPPRGA VCRLHSAASS LFPSLSGPQA GHQVGDSGAQ EARLPRTLGC GLGSPLHQPH
Gallus gal    PAR----HSN VKKVSGVGGT TYEISV---- ---------- ---------- ----------
Calidris p    PAR----HSN VKKVSGVGGT TYEISV---- ---------- ---------- ----------
Anas platy    PAR----HSN VKKVSGVGGT TYEISV---- ---------- ---------- ----------

Homo sapie    --
Sus scrofa    LD
Gallus gal    --
Calidris p    --
Anas platy    --
```

Amino acid alignment of DHHC8 of humans (homo sapiens), pigs (sus scrofa) and three avian species, chicken (gallus gallus), sandpiper (Calidris pugnas) and mallard duck (Anas platyrhynchos). The transmembrane regions and the amphiphilic helix following TMR4 are highlighted in grey. Cys and His residues that coordinate zinc ions, the DHHC motif and the TTEX motiv are highlighted in light blue. Most of the C-terminal region is predicted to be unstructured (underlined). Amino acid differences to human DHHC2 are highlighted in yellow in the sequence of the respective species.

Table S11: Amino acid differences between human DHHC20 and DHHC20 of animal hosts of Flu A

```
              ....|....| ....|....| ....|....| ....|....| ....|....| ....|....|
                      5         15         25         35         45         55
Homo sapie    MAPWTLWRCC QRVVGWVPVL FITFVVVWSY YAYVVELCVF TIFGNEENGK TVVYLVAFHL
Sus scrofa    MAPCTLWRCC QRSVGWVPVL FITFVVVWSY YAYVVELCVF TLSGNGENGK AVVYLVAFHL
Gallus gal    MAPSHVLKCC QRVLAWVPVV FIALVVAWSY YAYVVELCVF TISSTAEK-- -VVYLVIFHL
Calidris p    MAPSHVLKCC QKVLAWVPVA FIALVVAWSY YAYVVELCMF TISSTAEK-- -VVYLVVFHL
Anas platy    MAPPR-VKCC QKVLSWLPVV FIALVVAWSY YAYVVELCLF TISSTAEK-- -VVYLVVFHL
                                    TMR1                                 TMR2

              ....|....| ....|....| ....|....| ....|....| ....|....| ....|....|
                     65         75         85         95        105        115
Homo sapie    FFVMFVWSYW MTIFTSPASP SKEFYLSNSE KERYEKEFSQ ERQQEILRRA ARALPIYTTS
Sus scrofa    FFVMFVWSYW MTIFTSPASP SKEFYLSSSE KERYEKEFSQ ERQQEILRRA ARGLPVYTTS
Gallus gal    SFVMFVWSYG KTIFTSPASP SNEFCLSKAD KEQYEKEERP ESQQEILRRA AKDLPIYTTT
Calidris p    SFVMFVWSYW KTIFTSPASP SNEFCLSKAD KEQYEKEERP ESQQEILRRA AKDLPIYTTT
Anas platy    SFVMFVWSYW KTIFTSPASP SNEFCLSKAD KEQYEKEERP EFQQEILRRA AKDLPICTTT
                               Cysteine-rich domain

              ....|....| ....|....| ....|....| ....|....| ....|....| ....|....|
                    125        135        145        155        165        175
Homo sapie    ASKTIRYCEK CQLIKPDRAH HCSACDSCIL KMDHHCPWVN NCVGFSNYKF FLLFLLYSLL
Sus scrofa    ATQTIRYCER CQLIKPDRAH HCSACDICVL KMDHHCPWVN NCVGFSNYKF FLLFLLYSLL
Gallus gal    ASRAIRYCDR CQLIKPDRCH HCSACDICVL KMDHHCPWVN NCVGFSNYKF FLLFLMYSLL
Calidris p    ASRAIRYCDR CQLIKPDRCH HCSACDICVL KMDHHCPWVN NCVGFSNYKF FLLFLMYSLL
Anas platy    ASRAIRYCDR CQLIKPDRCH HCSACDICVL KMDHHCPWVN NCVGFSNYKF FLLFLMYSLL
              Acyl-CoA  Zinc-binding               DHHC                  TMR3

              ....|....| ....|....| ....|....| ....|....| ....|....| ....|....|
                    185        195        205        215        225        235
Homo sapie    YCLFVAATVL EYFIKFWTNE LTDTRAKFHV LFLFFVSAMF FISVLSLFSY HCWLVGKNRT
Sus scrofa    YCLFVATTVL QYFIKFWTNE LSDTRAKFHV LFLFFVSTMF FISVLSLLSY HCWLVGKNRT
Gallus gal    YCLFVAATVL QYFIKFWTNE LPDTHAKFHV LFLFFVAAMF FISILSLFSY HCWLVGKNRS
Calidris p    YCLFVAATVL QYFIKFWTNE LPDTHAKFHV LFLFFVAAMF FISILSLFSY HCWLVSKNRS
Anas platy    YCLFVAATVL QYFIKFWTKE LTDAYAKYHI LCLFFVAAIA FIGIQWLFSY HCWLVGKNRS
                                                      TMR4

              ....|....| ....|....| ....|....| ....|....| ....|....| ....|....|
                    245        255        265        275        285        295
Homo sapie    TIESFRAPTF SYGPDGNGFS LGCSKNWRQV FGDEKKYWLL PIFSSLGDGC SFPTRLVGMD
Sus scrofa    TIESFRAPTF SYGPDGNGFS LGCSKNWRQV FGDEKKYWLL PVFSSQGDGC SFPTRLVGTD
Gallus gal    TIETFRAPTF RNGPDKNGFS LGCSKNLREV FGDEKKYWLL PIFTSLGDGC NFPTRLVIMD
Calidris p    TIETFRAPTF RNGPDKNGFS LGCSKNLREV FGDEKKYWLL PIFTSLGDGC NFPTRLVIMD
Anas platy    TIESHRAPMF RNGPDENGFS LGYGKNLREV FGDEKKYWLL PIFTSLGDGC NFPTRLVIMD
              TTXE                          amphiphilic helix

              ....|....| ....|....| ....|....| ....|....| ....|....| ....|....|
                    305        315        325        335        345        355
Homo sapie    PEQASVTNQN EYARSSGSNQ PFPI-KPLSE SKNRLLDSES QWLENGAEEG IVKSGTNNHV
Sus scrofa    PEQASVSNQS ESGRSIGSNQ PFPI-KPLSE SKNRLLDSES QWLEDGAEEG VVRPGTNNHV
Gallus gal    PEQVTVSSQN EPAKSSGANQ PFPPTRPLSE SQNRLLASDS QWVESGPEDE NVKSGTKKHI
Calidris p    PEQLAVSSQN EPAKSSGANQ TFPP-RPLSE SQNRLLANDS QWVESGPEEE NVKSGAKKHI
Anas platy    PERVTVSTQT EPAKSSGASQ PFPP-QPLSE SQVRLLASDD QWVEIDPEEE NVK-------

              ....|.
                    365
Homo sapie    TVAIEN
Sus scrofa    TVAVEN
Gallus gal    IVSLES
Calidris p    IVSLES
Anas platy    ------
```

Amino acid alignment of DHHC20 of humans (homo sapiens), pigs (sus scrofa) and three avian species, chicken (gallus gallus), sandpiper (Calidris pugnas) and mallard duck (Anas platyrhynchos). The transmembrane regions are highlighted in grey, individual residues that contact the acyl chain in the cavity are highlighted in green and the two (S and T) that seal the cavity are highlighted in pink. The amphiphilic helix following TMR4 is also highlighted in grey. Cys and His residues that coordinate zinc ions, the DHHC motif and the TTEX motif are highlighted in light blue and basic residues involved in acyl-CoA binding are highlighted in green. LVGM is the end of the resolved DHHC20 structure. Amino acid differences to human DHHC2 are highlighted in yellow in the sequence of the respective species and the non-conservative exchanges labeled in the 3D-structure of DHHC20 are in red.

Table S12: Amino acid differences between human DHHC8 and DHHC8 from animal hosts of β-CoVs

```
                              10        20        30        40        50        60        70
                      ....|....|....|....|....|....|....|....|....|....|....|....|....|....|
Human                 MPRSPGTRLKPAKYIPVATAAALLVGSSTLFFVFT-----------------------------------
Rhinolophus Sinicus   MPRSPGTRLKPAKYIPVATAAALLVGSSTLFFVFT-----------------------------------
Pipistrellus Kuhlii   MPRSPGTRLKPAKYIPVATAAALLIGSSTLFFVFT-----------------------------------
Camelus Dromedarius   MPRSPGTRLKPAKYIPVATAAALLVGSSTLFFVFT-----------------------------------
Manis Javanica        MPRSPGTRLKPAKYIPVATAAALLVGSSALFFVFTLTWALLEALTVRPTSDFRGGRMSLSLTRPQAAGPE
                                             TMR1

                              80        90       100       110       120       130       140
                      ....|....|....|....|....|....|....|....|....|....|....|....|....|....|
Human                 ---------------------------CPWLTRAVSPAVPVYNGIIFLFVLANFSMATFMDPGVFPRADE
Rhinolophus Sinicus   ---------------------------CPWLTRAVSPAVPVYNGIIFLFVLANFSMATFMDPGIFPRADE
Pipistrellus Kuhlii   ---------------------------CPWLTHAVSPAVPVYNGIIFLFVLANFSMATFMDPGVFPRADE
Camelus Dromedarius   ---------------------------CPWLTRAVSPAVPVYNGILFLFVLANFSMATFMDPGVFPRADE
Manis Javanica        AAAPCQVPSPPSALLTAGALGSGTPALCPWLTRAVSPAVPVYNGIIFLFVLANFSMATFMDPGVFPRADE
                                                                             TMR2

                             150       160       170       180       190       200       210
                      ....|....|....|....|....|....|....|....|....|....|....|....|....|....|
Human                 DEDKEDDFRAPLYKNVDVRGIQVRMKWCATCHFYRPPRCSHCSVCDNCVEDFDHHCPWVNNCIGRRNYRY
Rhinolophus Sinicus   DEDKEDDFRAPLYKNVDVRGIQVRMKWCATCHFYRPPRCSHCSVCDNCVEDFDHHCPWVNNCIGRRNYRY
Pipistrellus Kuhlii   DEDKEDDFRAPLYKNVDVRGIQVRMKWCATCHFYRPPRCSHCSVCDNCVEDFDHHCPWVNNCIGRRNYRY
Camelus Dromedarius   DEDKEDDFRAPLYKNVDVRGIQVRMKWCATCHFYRPPRC-PLQRCDNCAQDFDHHCPWVNNCIGRRNYRY
Manis Javanica        DEDKEDDFRAPLYKNVDVRGIQVRMKWCATCHFYRPPRCSHCSVCDSCVEDFDHHCPWVNNCIGRRNYRY
                                                 Zinc-binding            DHHC  DHHC domain

                             220       230       240       250       260       270       280
                      ....|....|....|....|....|....|....|....|....|....|....|....|....|....|
Human                 FFLFLLSLSAHMVGVVAFGLVYVLNHAEGLGAAHTTITMAVMCVAGLFFIPVIGLTGFHVVLVTRGRTTN
Rhinolophus Sinicus   FFLFLLSLSAHMVGVVAFGLVYVLNHAEGLGAAHTTITMAVMCVAGLFFIPVIGLTGFHVVLVTRGRTTN
Pipistrellus Kuhlii   FFLFLLSLSAHMVGVVAFGLVYVLNHAEGLGAAHTTITMAVMCVAGLFFIPVIGLTGFHVVLVTRGRTTN
Camelus Dromedarius   FFLFLLSLSAHMVGVVAFGLVYVLHHAEGLGAAHTTITMAVMCVAGLFFIPVIGLTGFHVVLVTRGRTTN
Manis Javanica        FFLFLLSLSAHMVGVVAFGLVYVLNHAEGLGATHTTVTMAVMGVAGLFFIPVIGLTGFHVVLVTRGRTTN
                              TMR3                                          TMR4          TTXE

                             290       300       310       320       330       340       350
                      ....|....|....|....|....|....|....|....|....|....|....|....|....|....|
Human                 EQVTGKFRGGVNPFTRGCCGNVEHVLCSPLAPRYVVEPP-RLPLAVSLKPPFLRPELLDRAAPLKVKLSD
Rhinolophus Sinicus   EQVTGKFRGGVNPFTRGCYGNVEHVLCSPLAPRYVVEPP-RLPLAARLKPPFFRPELLERAAPLKVKLSD
Pipistrellus Kuhlii   EQVTGKFRGGVNPFTRGCYGNVEHVLCSPLAPRYVVEPPPRLPLAARLKPPFLRPELLDRAPPLKVKLSD
Camelus Dromedarius   EQVTGKFRGGVNPFTRGCYGNVEHVLCSPLAPRYVVRAA-RLPLAARLKPPFLRPELLERAAPLKVKLSD
Manis Javanica        EQVTGKFRGGVNPFTRGCYGNVEHVLCSPLAPRYVVEPP-QLPLATRLKPPFLRPELLGRAAPLKVKLSD
                                           amphiphilic helix
```

```
                            360       370       380       390       400       410       420
                     ....|....|....|....|....|....|....|....|....|....|....|....|....|....|
Human                NGLKAGLGRSKSKGSLDRLDEKPLDLGPPLPPKIEAGTFSSDLQTPRPGSAESALSVQRTSPPTPAMYKF
Rhinolophus Sinicus  NGLKASLGHSKSKGSLDRLDEKPLDLGPPLPPKAEAVTFSGDRQTPRPGSAESTLSAQRTSPPTPAMYKF
Pipistrellus Kuhlii  NGLKAGLGRSKSKGSLDRLDEKPLDLGPPLPPKPEAGTFGGDRQTPRPGSAESALSAQRTSPPTPAMYKF
Camelus Dromedarius  NGLKAGLGRSKSKGSLDRLDEKPLDLGPPLPPKAEAGTFASDLQTPRPGSAESALSAQRTSPPTPAMYKF
Manis Javanica       NGLKASLGRSKSKGSLDRLDEKPLDLGPPLPSKVEVGTFGSNLQTPRPGSAESALKVQRTSPPTPAMYKF

                            430       440       450       460       470       480       490
                     ....|....|....|....|....|....|....|....|....|....|....|....|....|....|
Human                RPAFPTGPKVPFCGPGEQVPGPDSLTLGDDSIRSLDFVSEPSLDLPDYGPGG-LHAAYPPSPPLSASDAF
Rhinolophus Sinicus  RPTFPMGPKAPFCGPGEQVPGPDSLALGEDSIHSLDFTSEPNLDLPDYTPGG-LHTAYPPSPPLSASDTF
Pipistrellus Kuhlii  RPAFPAGPKAPFCGPGEQVSGPDSLTLGEDSIHSLDFTSEPSLDLPDFAAGGGLHAPYAPSPPLGAADTF
Camelus Dromedarius  RPAFPTGPKAPFCGPGEQVTGPDSLTLGEDSIHSLDFASEPSLDLPDYPPSG-LHAAYPPSPPLSATDTF
Manis Javanica       RPAFPMGPKSPFCGPGEQVPGPDSLTLGEDSVHGLDFTSEPHLDLPDYPPGG-LHAAYPPSPQLSVADTF

                            500       510       520       530       540       550       560
                     ....|....|....|....|....|....|....|....|....|....|....|....|....|....|
Human                SGALRSLSLKASSRRGGDHVALQPLRSEGGPPTPHRSIFAPHALPNRNGSLSYDSLLNPGSPGGHACPAH
Rhinolophus Sinicus  SGALRSLSLKAASRRGGDHIALQSLRSEGGPPTPHHSIFAPHALSNRNGSLSYDSLLNPGSPSGHACPAH
Pipistrellus Kuhlii  SGALRSLSLKAAGRRGGDHVALQPLRSEGGPPTPHRGVFAPHALPNRNGSLSYDSLLSPASPGGRACPAH
Camelus Dromedarius  SGALRSLSLKAASRRGGDHMALQPLRSEGGPPTPHRSIFPSHALPNRNGSLSYDSLLNPGSPGGHTCPAH
Manis Javanica       SGALRSLSLKAAGRRGRDHVALQPLRSEGGPPTPHRGIFAPHALPNRNGSLSYDSLLDPGSPGGHACPAH

                            570       580       590       600       610       620       630
                     ....|....|....|....|....|....|....|....|....|....|....|....|....|....|
Human                PAVG-VAGYHSPYLHPGATGDPPRPLPRSFSPVLGPRPREPSPVRYDNLSRTIMASIQERKDREERERLL
Rhinolophus Sinicus  PSAG-MASYRSPYLHPGAVGDPPRPPPRSFSPVLGPRPREPSPVRYDNLSRTIMASIQERKDREERERLL
Pipistrellus Kuhlii  PAAGGLAGYRSPYLHP---GDPPRPPPRSFSPVLGPRPREPSPVRYDNLSRTIMASIQERKDREERERLL
Camelus Dromedarius  PSAG-VAGYRAPYLHAGAVGDPPRPPPRSFSPVLGPRPREPSPVRYDNLSRTIMASIQERKDREERERLL
Manis Javanica       PPAG-MASYRSPYLHPGSVGDPPRPPPRSFSPVLGSRPREPSPVRYDNLSRTIMASIQERKDREEHQRLL

                            640       650       660       670       680       690       700
                     ....|....|....|....|....|....|....|....|....|....|....|....|....|....|
Human                RSQADSLFGDSGVYDAPSSYSLQQAS-VLSEGPRGPALRYGSRDDLVAGPG---FGGARNPALQTSLSSL
Rhinolophus Sinicus  RSQADSLFGDSGVYDAPSSYSLQQAS-VLSEGPRGPALHYGSRDDLVAGPS---FRGARNPALQTSLSSL
Pipistrellus Kuhlii  RSQADSLFGDAGAYDAPGSYGLQQAAGALSEGPRGPAARYGSRDDLVAGPGGAGFGGARNPALQASVTSL
Camelus Dromedarius  RSQADSLFGDSGVYDAPSSYSLQQAS-VLSEGPRGPALRYGSRDDLVAGPG---FGGARNPALQASLSSL
Manis Javanica       RSQADSLFGDSGVYDAPSSYSLQQAG-VLAEGPRGPVLRCGSRDDLVAGPG---FGGARNPALQTSLSSL

                            710       720       730       740       750       760       770
                     ....|....|....|....|....|....|....|....|....|....|....|....|....|....|
Human                S-----SSVSRAPRTSSSSLQADQASSNAPGPRPSSGSHRSPARQGLPSPP-GTPHSPSYAGPKAVAFIH
Rhinolophus Sinicus  S-----SAISRAPRTSSSSLQADLANNNAPGPRPGSASQRSPLRQGPPSPP-STPRSPTFMGPKAVAFIH
Pipistrellus Kuhlii  SGVAAAAAGSRAPRTSSSSLQADLANNNAPGPRPGSGPHRSPLRQGAPSPPPSTPRSPSYVGPKAVAFIH
Camelus Dromedarius  S-----SAVSRAPRTSSSSLQADLTNSSAPGTRPPSGSHRSPARQVPPSPP-GTPRSPSYGGPKAVAFIH
Manis Javanica       S-----SAVSRAPRTSSSSLQADLANSNAPVPRPSSGSHRSPARQGLPSPP-GTPCSPSYPGPKAVAFVH
```

```
                                    780       790       800       810       820       830       840
                           ....|....|....|....|....|....|....|....|....|....|....|....|....|....|
Human                      TDLPEPPPSLTVQ---------------------------------------------------------
Rhinolophus Sinicus        TDLPEPPPSLAVQ-------------------------------------------------------RG
Pipistrellus Kuhlii        SDLPEPPPSLAVQ---------------------------------------------------------
Camelus Dromedarius        TDLPEPPPSLAMQ-------------------------------------------------------RG
Manis Javanica             TDLPESPPSLAVQSLCRPRASSSGDGGTDPVQENESGGHNPAANIPWVPGPWSRLGRTGPGSTRFPAQRG

                                    850       860       870       880       890       900       910
                           ....|....|....|....|....|....|....|....|....|....|....|....|....|....|
Human                      ------RDHPQLKTP--PSKLNGQSPGLARLGPATGPPGPSAS--PTRHTLVKKVSGVGGTTYEISV---
Rhinolophus Sinicus        RIGTCSRGWGRRGPPRVPPGLHLCHLGLPEDRPPLRVPWSPAAGVSPRGAVCRLHLAASSLFPRFSGPER
Pipistrellus Kuhlii        ------RDHPQLKNP--PSKLNGQSPGLARLGPTAGPPGPVTS--PARHTLVKKVSGVGGTTYEISV---
Camelus Dromedarius        WTGTCSRGWGRRGQPRAPPGLHLCHLGLPEDRPALRASWSPAAGAPYRGAVCRLHSAASSLFPSLSGPER
Manis Javanica             RIGTCSRGWGRRGQPWAPPGLHLCHLGLPEDRPPLQAPRSPATRMPSRGAVCRLHSAASSLFTGLSGPER
                                                                               PDZ ligand

                           ...
Human                      ---
Rhinolophus Sinicus        DTK
Pipistrellus Kuhlii        ---
Camelus Dromedarius        DTK
Manis Javanica             DTQ
```

Amino acid alignment of DHHC8 proteins from humans (homo sapiens), camel (camelus dromedaries, pangolin (manis javanica) and two bat species, Rhinolophus Sinicus (host of various β-CoVs including SARS-1 and presumably SARS-2) and Pipistrellus Kuhlii. The transmembrane regions and the amphiphilic helix following TMR4 are highlighted in grey. Cys and His residues that coordinate zinc ions, the DHHC motif and the TTEX motiv are highlighted in light blue. Most of the C-terminal region is predicted to be unstructured (underlined). Amino acid differences to human DHHC8 are highlighted in yellow in the sequence of the respective species.

Accession numbers: DHHC8 sequences of Human (NP_037505.1), Rhinolophus Sinicus (XP_019611479), Pipistrellus Kuhlii (XP_036305544.1), Camelus_Dromedarius (XP_031299130) and Manis Javanica (XP_036849239.1)

Table S13: Amino acid differences between human DHHC9 and DHHC9 from animal hosts of β-CoVs

```
                             10        20        30        40        50        60        70
                     ....|....|....|....|....|....|....|....|....|....|....|....|....|....|
Human                MSVMVVRKKVTRKWEKLPGRNTFCCDGRVMMARQKGIFYLTLFLILGTCTLFFAFECRYLAVQLSPAIPV
Rhinolophus Sinicus  MSVIVVRKKVTRKWEKLPGRNTFCCDGRVMMARQKGIFYLTLFLILGTCTLFFAFECRYLAVQLSPAIPV
Pipistrellus Kuhlii  MSVMVVRKKVTRKWEKLPGRNTFCCDGRVMMARQKGIFYLTLFLILGTCTLFFAFECRYLAVQLSPAIPV
Camelus Dromedarius  MSVMVVRKKVTRKWEKLPGRNTFCCDGRVMMARQKGIFYLTLFLILGTCTLFFAFECRYLAVQLSPAIPV
Manis Javanica       MSVMVVRKKVTRKWEKLPGRNTFCCDGRVMMARQKGIFYLTLFLILGTCTLFFAFECRYLAVQLSPAIPV
                                                                 TMR1

                             80        90       100       110       120       130       140
                     ....|....|....|....|....|....|....|....|....|....|....|....|....|....|
Human                FAAMLFLFSMATLLRTSFSDPGVIPRALPDEAAFIEMEIEATNGAVPQGQRPPPRIKNFQINNQIVKLKY
Rhinolophus Sinicus  FAAMLFLFSMATLLRTSFSDPGVIPRALPDEAAFIEMEIEATNGTVPQGQRPPPRIKNFQINNQIVKLKY
Pipistrellus Kuhlii  FAAMLFLFSMATLLRTSFSDPGVIPRALPDEAAFIEMEIEATNGAVPQGQRPPPRIKNFQINNQIVKLKY
Camelus Dromedarius  FAAMLFLFSMATLLRTSFSDPGVIPRALPDEAAFIEMEIEATNGAVPQGQRPPPRIKNFQINNQIVKLKY
Manis Javanica       FAAMLFLFSMATLLRTSFSDPGVIPRALPDEAAFIEMEIEATNGAVPQGQRPPPRIKNFQINNQIVKLKY
                         TMR2          cysteine-rich domain

                            150       160       170       180       190       200       210
                     ....|....|....|....|....|....|....|....|....|....|....|....|....|....|
Human                CYTCKIFRPPRASHCSICDNCVERFDHHCPWVGNCVGKRNYRYFYLFILSLSLLTIYVFAFNIVYVALKS
Rhinolophus Sinicus  CYTCKIFRPPRASHCSICDNCVERFDHHCPWVGNCVGKRNYRYFYLFILSLSLLTIYVFAFNIVYVALKS
Pipistrellus Kuhlii  CYTCKIFRPPRASHCSICDNCVERFDHHCPWVGNCVGKRNYRYFYLFILSLSLLTIYVFAFNIVYVALKS
Camelus Dromedarius  CYTCKIFRPPRASHCSICDNCVERFDHHCPWVGNCVGKRNYRYFYLFILSLSLLTIYVFAFNIVYVALKS
Manis Javanica       CYTCKIFRPPRASHCSICDNCVERFDHHCPWVGNCVGKRNYRYFYLFILSLSLLTIYVFAFNIVYVALKS
                              Zinc-binding    DHHC                       TMR3

                            220       230       240       250       260       270       280
                     ....|....|....|....|....|....|....|....|....|....|....|....|....|....|
Human                LKIGFLETLKETPGTVLEVLICFFTLWSVVGLTGFHTFLVALNQTTNEDIKGSWTGKNRVQNPYSHGNIV
Rhinolophus Sinicus  LKIGFLETLKETPGTVLEVLICFFTLWSVVGLTGFHTFLVALNQTTNEDIKGSWTGKNRVQNPYSHGNIV
Pipistrellus Kuhlii  LKIGFLETLKETPGTVLEVLICFFTLWSVVGLTGFHTFLVALNQTTNEDIKGSWTGKNRVQNPYSHGNIV
Camelus Dromedarius  LKIGFLETLKETPGTVLEVLICFFTLWSVVGLTGFHTFLVALNQTTNEDIKGSWTGKNRVQNPYSHGNIV
Manis Javanica       LKIGFLETLKETPGTVLEVLICFFTLWSVVGLTGFHTFLVALNQTTNEDIKGSWTGKNRVQNPYSHGNIV
                                              TMR4               TTXE

                            290       300       310       320       330       340       350
                     ....|....|....|....|....|....|....|....|....|....|....|....|....|....|
Human                KNCCEVLCGPLPPSVLDRRGILPLEESGSRPPSTQETSSSLLPQSPAPTEHLNSNEMPEDSSTPEEMPPP
Rhinolophus Sinicus  KNCCEVLCGPLPPSVLDRRGILPLEESGSRPPSTQETSSSLLPQSPALTEHLSTNKMPEDTSTPAKMPPP
Pipistrellus Kuhlii  KNCCEVLCGPLPPSVLDRRGILPLEESGSRPPSTQETSSSLLPQSPVSVPDFKILRALTEHLSSNEMPED
Camelus Dromedarius  KNCCEVLCGPLPPSVLDRRGILPLEESGSRPPSTQETSSSLLPQSPVPTDHLSSSEMPEDTSTPEEMPPP
Manis Javanica       KNCCEVLCGPLPPSVLDRRGILPLEESGSRPPSTQETSSSLLPQNPAPTEHLSSNEMPEDTSTPEEMPPP
                     amphiphilic helix                   Pro-rich unstructured region
```

```
                           360       370
                     ....|....|....|....|...
Human                ---------EPPEPPQEAAEAEK
Rhinolophus Sinicus  ---------EPPEPPQEVAEADK
Pipistrellus Kuhlii  STPEEMPPPEPPEPPQEVAEADK
Camelus Dromedarius  ---------EPPEPPQEGTEAEK
Manis Javanica       ---------EPPEPPQEMTEAEK
```

Amino acid alignment of DHHC9 proteins from humans (homo sapiens), camel (camelus dromedaries, pangolin (manis javanica) and two bat species, Rhinolophus Sinicus (host of various β-CoVs including SARS-1 and presumably SARS-2) and Pipistrellus Kuhlii, host of MERS. The transmembrane regions and the amphiphilic helix following TMR4 are highlighted in grey. Cys and His residues that coordinate zinc ions, the DHHC motif and the TTEX motiv are highlighted in light blue. Most of the C-terminal region is predicted to be unstructured (underlined). Amino acid differences to human DHHC9 are highlighted in yellow in the sequence of the respective species.

Accession numbers: DHHC9 sequences of Human (NP_001008223.1), Rhinolophus Sinicus (XP_019572571.1), Pipistrellus Kuhlii (XP_036299136.1), Camelus Dromedarius (XP_031301040.1) and Manis Javanica (XP_036872770.1)

Table S14: Amino acid differences between human DHHC20 and DHHC20 from animal hosts of β-CoVs

```
                                   10        20        30        40        50        60        70
                           ....|....|....|....|....|....|....|....|....|....|....|....|....|....|
Human                      MAPWTLWRCCQRVVGWVPVLFITFVVVWSYYAYVVELCVFTIFGNEENGKTVVYLVAFHLFFVMFVWSYW
Rhinolophus Sinicus        MAPGRFWRCCQRGVGWVPVLFIAFVVAWSYYAYVVELCVFTLSGNGENGKAVVYLVTFHLSFVMFVWSYW
Pipistrellus Kuhlii        MAPGRFWRCCLRGAGWVPVLFITFVAAWSYYAYVVELCLFTLSGNGENGKAVAYLVAFHLFFVMFVWSYW
Camelus Dromedarius        MAPGRFWRCCQRGVGWVPVLFIAFVVAWSYYAYVVELCVFTLSGNGENGKAVVYLVTFHLSFVMFVWSYW
Manis Javanica             ---------------------------------------------------------------MFVWSYW
                                                TMR1                                    TMR2

                                   80        90       100       110       120       130       140
                           ....|....|....|....|....|....|....|....|....|....|....|....|....|....|
Human                      MTIFTSPASPSKEFYLSNSEKERYEKEFSQERQQEILRRAARALPIYTTSASKTIRYCEKCQLIKPDRAH
Rhinolophus Sinicus        MTIFTTPASPSKEFYLSSSEKERYEKEFSPERQQDILRRTARDLPIYTTSGSRTIRYCERCQLIKPDRAH
Pipistrellus Kuhlii        MTIFTSPATPSKEFYLSNSEREHYEKEFSQERQQEILRRTAKDLPIYTTSGSRTIRYCERCQLIKPDRAH
Camelus Dromedarius        MTIFTSPASPSKEFYLSSSEKERYEKEFSPERQQDILRRTARDLPIYTTSGSRTIRYCERCQLIKPDRAH
Manis Javanica             MTIFSSHASPSKEFYLSNSEKEHYEKEFSQERQQEILRRAARDLPIYTTSASRTIRYCERCQLIKPDRAH
                                  Cysteine-rich domain              Acyl-CoA         Zinc-binding

                                  150       160       170       180       190       200       210
                           ....|....|....|....|....|....|....|....|....|....|....|....|....|....|
Human                      HCSACDSCILKMDHHCPWVNNCVGFSNYKFFLLFLLYSLLYCLFVAATVLEYFIKFWT------------
Rhinolophus Sinicus        HCSACDICILKMDHHCPWVNNCVGFSNYKFFLLFLLYSLLYCLFVATTVLQYFIKFWTLCRRKSTESCPQ
Pipistrellus Kuhlii        HCSACDICILKMDHHCPWVNNCVGFSNYKFFLLFLLYSLLYCLFVATTVLQYFIKFWTLCHRKSTQSCPK
Camelus Dromedarius        HCSACDICILKMDHHCPWVNNCVGFSNYKFFLLFLLYSLLYCLFVATTVLQYFIKFWTLCRRKSTESCPQ
Manis Javanica             HCSACDICILKMDHHCPWVNNCVGFSNYKFFLLFLLYSLLYCLFVAATVLQYFIKFWTLCHRKSTESCPK
                                      DHHC                   TMR3

                                  220       230       240       250       260       270       280
                           ....|....|....|....|....|....|....|....|....|....|....|....|....|....|
Human                      NELTDTRAKFHVLFLFFVSAMFFISVLSLFSYHCWLVGKNRTTIESFRAPTFSYGPDGNGFSLGCSKNWR
Rhinolophus Sinicus        NELPDTRAKFHVLFLFFVSTMFFISVLSLFSYHCWLVGKNRTTIESFRAPTFSYGQDGNGFSLGYSKNWR
Pipistrellus Kuhlii        NELTDTRAKFHVLFLFFVSTMFFISVLSLFSYHCWLVGKNRTTIESFSAPTFSYGPDGNGFSLGYSKNWR
Camelus Dromedarius        NELPDTRAKFHVLFLFFVSTMFFISVLSLFSYHCWLVGKNRTTIESFRAPTFSYGQDGNGFSLGHSKNWR
Manis Javanica             NELSDTRAKFHILFLFFVSAMFFVSVLSLFSYHCWLVGRNRTTIESFRAPTFSYGPDGNGFSLGYSKNWR
                                              TMR4                TTXE

                                  290       300       310       320       330       340       350
                           ....|....|....|....|....|....|....|....|....|....|....|....|....|....|
Human                      QVFGDEKKYWLLPIFSSLGDGCSFPTRLVGMDPEQASVTNQNEYARSSGSNQPFPIKPLSESKNRLLDSE
Rhinolophus Sinicus        QVFGDEKKYWLLPIFSSLGDGCSFPTRLVGMDPEQASVTNQNEYARSIGSNPPFPIRPLSESKNRLLDSE
Pipistrellus Kuhlii        QVFGDEKKYWLLPIFSSLGDGCSFPTRLVGMDPEQASVTNQNEYARSIGSNQPFPIKPLSESKNRLLDSD
Camelus Dromedarius        QVFGDEKKYWLLPIFSSLGDGCSFPTRLVGMDPEQASVTNQNEYARSIGSNPPFPVRPLSESKNRLLDSE
Manis Javanica             QVFGDEKKYWLLPIFSSLGDGCSFPARLVGTDPERASVTSQNECARSISSNQPFPIKPLSESKNRLLDSE
```

```
                                                          360       370       380       390
                                                  ....|....|....|....|....|....|....|....|...
Human                                             SQWLENGAEEGIVKSGTNNHVTVAIEN----------------
Rhinolophus Sinicus                               CQWTENGSEEGTVRSGTNNHVTVAIEN----------------
Pipistrellus Kuhlii                               SQWTENGSEEDTIRSGINNFVTVAIDKKVTCPLLKKLDPEKSD
Camelus Dromedarius                               CQWTENGSEEGTVRSGTNNHVTVAIQN----------------
Manis Javanica                                    SHWIENGSEEGIVRSGTNNHVTVAIEN----------------
```

Amino acid alignment of DHHC20 proteins from humans (homo sapiens), camel (camelus dromedaries, pangolin (manis javanica) and two bat species, Rhinolophus Sinicus (host of various β-CoVs including SARS-1 and presumably SARS-2) and Pipistrellus Kuhlii, host of MERS. The transmembrane regions are highlighted in grey, individual residues that contact the acyl chain in the cavity are highlighted in green and the two (S and T) that seal the cavity are highlighted in pink. The amphiphilic helix following TMR4 is also highlighted in grey. Cys and His residues that coordinate zinc ions, the DHHC motif and the TTEX motiv are highlighted in light blue and basic residues involved in acyl-CoA binding are highlighted in green. LVGM is the end of the resolved DHHC20 structure. Amino acid differences to human DHHC2 are highlighted in yellow in the sequence of the respective species and the non-conservative exchanges labeled in the 3D-structure of DHHC20 are in red.

Accession numbers: DHHC20 sequences of Human (NP_001316988.1), Rhinolophus Sinicus (XP_019571223.1), Pipistrellus Kuhlii (XP_036313463.1), Camelus Dromedarius (XP_031321583.1) and Manis Javanica (XP_036857811.1).
